# D-Ribose Interferes with Quorum Sensing to Inhibit Biofilm Formation of *Lactobacillus paraplantarum* L-ZS9

**DOI:** 10.3389/fmicb.2017.01860

**Published:** 2017-09-26

**Authors:** Lei Liu, Ruiyun Wu, Jinlan Zhang, Nan Shang, Pinglan Li

**Affiliations:** ^1^Beijing Advanced Innovation Center for Food Nutrition and Human Health, College of Food Science and Nutritional Engineering, China Agricultural University, Beijing, China; ^2^Key Laboratory of Functional Dairy, China Agricultural University, Beijing, China; ^3^Department of Agricultural, Food and Nutritional Sciences, University of Alberta, Edmonton, AB, Canada

**Keywords:** *Lactobacillus paraplantarum* L-ZS9, quorum sensing inhibitor, biofilms, proteomics, recombinant strains

## Abstract

Biofilms help bacteria survive under adverse conditions, and the quorum sensing (QS) system plays an important role in regulating their activities. Quorum sensing inhibitors (QSIs) have great potential to inhibit pathogenic biofilm formation and are considered possible replacements for antibiotics; however, further investigation is required to understand the mechanisms of action of QSIs and to avoid inhibitory effects on beneficial bacteria. *Lactobacillus paraplantarum* L-ZS9, isolated from fermented sausage, is a bacteriocin-producing bacteria that shows potential to be a probiotic starter. Since exogenous autoinducer-2 (AI-2) promoted biofilm formation of the strain, expression of genes involved in AI-2 production was determined in *L. paraplantarum* L-ZS9, especially the key gene *luxS*. D-Ribose was used to inhibit biofilm formation because of its AI-2 inhibitory activity. Twenty-seven differentially expressed proteins were identified by comparative proteomic analysis following D-ribose treatment and were functionally classified into six groups. Real-time quantitative PCR showed that AI-2 had a counteractive effect on transcription of the genes *tuf, fba, gap, pgm, nfo, rib*, and *rpoN*. Over-expression of the *tuf, fba, gap, pgm*, and *rpoN* genes promoted biofilm formation of *L. paraplantarum* L-ZS9, while over-expression of the *nfo* and *rib* genes inhibited biofilm formation. In conclusion, D-ribose inhibited biofilm formation of *L. paraplantarum* L-ZS9 by regulating multiple genes involved in the glycolytic pathway, extracellular DNA degradation and transcription, and translation. This research provides a new mechanism of QSI regulation of biofilm formation of *Lactobacillus* and offers a valuable reference for QSI application in the future.

## Introduction

Bacteria live in dense and diverse communities termed biofilms, which is a major mode of microbial life (Nadell et al., 2016). Biofilms are sessile microbial communities that attach to biotic and abiotic surfaces and survive as self-organized, three-dimensional structures by producing an extracellular polymeric matrix (Penesyan et al., 2015). This lifestyle helps microorganisms to survive in unfavorable environments (Taylor et al., 2014; Olsen, 2015). Biofilms of pathogenic bacteria may cause antibiotic-tolerant infections as well as damage to surfaces and flow systems (Hoiby et al., 2010; Bixler and Bhushan, 2012; Drescher et al., 2013). However, similar to some non-pathogenic microorganisms and beneficial bacteria, biofilms enable pathogenic bacteria to resist environmental conditions, leading to successful colonization and maintenance of their population (Lepargneur and Rousseau, 2002).

Within biofilms, bacteria use a quorum sensing (QS) system to communicate with each other. The LuxS/AI-2 QS system exists widely in both Gram-negative and Gram-positive bacteria. The signal molecule, autoinducer-2 (AI-2), has been deemed as a universal language for intra-species and inter-species communication (Schauder et al., 2001; Gospodarek et al., 2009). The *luxS* AI-2 synthase gene homolog has been found in a wide range of bacteria, with 17% of the phylum *Bacteroidetes* and 83% of the *Firmicutes* predicted to have the homolog according to the KEGG database (Thompson et al., 2015). Although most research has focused on the regulation of AI-2 in biofilms of pathogens, some non-pathogenic and beneficial bacteria have been reported to use this ‘universal’ and ‘common’ signaling system to regulate their behavior (Park et al., 2016). For example, AI-2 signaling has been reported to regulate cell growth and metabolism of *Lactobacillus* (Buck et al., 2009; Moslehi-Jenabian et al., 2009; Park et al., 2016). And in *Bifidobacteria*, AI-2 activity correlates with biofilm formation and gut colonization (Christiaen et al., 2014; Sun et al., 2014).

Since QS inhibitors (QSIs) hinder biofilm formation and reduce bacterial virulence in the biofilm state, they are considered as an ideal tool to inhibit the biofilm formation of pathogens and provide a good target to control bacterial infection (Pan and Ren, 2009; Harjai et al., 2014). An ideal QSI should significantly interfere with the QS system and social behavior without toxic effects on the bacteria and/or host. Ribose has no toxic effects and shares structural similarity with AI-2, which exhibits a furanosyl borate diether form (Cao and Meighen, 1989). This resemblance has been thought to cause competition between AI-2 and ribose. Thus, ribose has been used as a QSI to inhibit biofilm formation of various pathogenic bacteria (Armbruster et al., 2011; Lee et al., 2015; Cho et al., 2016; Ryu et al., 2016). However, the underlying mechanism of biofilm inhibition of non-pathogenic and beneficial bacteria still needs to be investigated.

Recently, LuxS/AI-2 QSIs have shown great potential to replace antibiotics to control pathogenic biofilm formation and infection (Brackman et al., 2009, 2011; Roy et al., 2013; Ryu et al., 2016). However, adequate considerations should be noted when QS therapy develops rapidly. Because of the common existence of LuxS/AI-2 QS in microorganisms, LuxS/AI-2 QSIs may inhibit biofilm formation of non-pathogenic and beneficial organisms as well. *Lactobacillus paraplantarum* L-ZS9 was originally isolated from fermented sausage and was proven to produce class IIb bacteriocins to inhibit the growth of pathogenic bacteria in our previous study (Wang et al., 2015; Zhang et al., 2016). It has the potential to be used as a probiotic starter. This study determined AI-2 production in L-ZS9 and AI-2 inhibitory activity by D-ribose. The effects of D-ribose on biofilm formation of L-ZS9 and its underlying mechanism were investigated and analyzed. This study provides new information about the regulation mechanism of this QSI on biofilm formation of *Lactobacillus* and has value as a reference for the reasonable application of QSI.

## Materials and Methods

### Bacterial Strains and Culture

*Lactobacillus paraplantarum* L-ZS9 was incubated at 37°C in de Man-Rogosa-Sharpe (MRS) broth (Bridge, Beijing) and MRS agar aerobically. *Escherichia coli* DH5α (Takara, Dalian) was incubated in Lennox broth or on solid medium with 1.5% (w/v) agar at 37°C. DH5α and L-ZS9 transformed with pMG76e vector were cultured with 200 and 3 μg/mL erythromycin, respectively. The pMG76e vector was provided by Professor Shangwu Chen from China Agricultural University (CAU, Beijing, China). *Vibrio harveyi* BB170 and BB152 were provided by Professor Xiangan Han from Shanghai Veterinary Research Institute (CAAS, Shanghai, China) and cultured in Marine Broth 2216 (Difco, United States) or Autoinducer Bioassay (AB) medium (Bassler et al., 1993).

### Analysis of Genes Related to AI-2 Production

The complete genome sequence of *L. paraplantarum* L-ZS9 is deposited in GenBank with accession number CP013130 (Liu and Li, 2016). The *pfs, luxS, metE*/*metH*, and *metK* genes involved in AI-2 production and the *sahH* gene responsible for metabolism of *S*-adenosylhomocysteine (SAH) to *S*-ribosylhomocysteine in one-step were searched for in the genome sequence of *L. paraplantarum* L-ZS9 according to their gene annotations by using the BLAST program of NCBI. Sizes of these genes were analyzed by Primer Input 3.0^1^, and locations were determined by analyzing the genome map.

### Growth-Curve Assay

To determine the effects of AI-2 and D-ribose on the growth of *L. paraplantarum* L-ZS9, a growth-curve assay was conducted. *L. paraplantarum* L-ZS9 was cultured in MRS broth in the presence or absence of AI-2 (3.7, 18.5, 37, 185 μM) (initial concentration 3.7 mM, purchased from OMM Scientific, United States). *L. paraplantarum* L-ZS9 was also cultured in MRS broth in the presence or absence of D-ribose (0, 10, 50, and 100 mM). Cultures were incubated at 37°C for 36 h. After 3, 6, 9, 12, 15, 18, 21, 24, 27, 30, 33, and 36 h, the optical density at 600 nm was determined using a UV-1800 Spectrophotometer (Shanghai Meipuda instrument, China).

### Determination of AI-2-Mediated Bioluminescence and AI-2 Inhibitory Activity of D-Ribose

*Lactobacillus paraplantarum* L-ZS9 was cultured in 12% (w/v) skim milk medium. After culture for 2, 4, 6, 8, 10, 12, 14, 16, 18, 20, 22, and 24 h, cells were collected by centrifuging at 12,000 *g*, 4°C, for 10 min. The cell-free culture fluid (CF) was obtained by filtering through a 0.22-μm filter (Millipore, Bedford, MA, United States) and adjusted to pH 7.0. The reporter strain *V. harveyi* BB170 was diluted 1:5000 with AB medium, and the CF sample was added to the diluted BB170 culture at 1:10 (v/v). The mixture was incubated at 28°C for 5 h, and 100 μL aliquots were added to white, flat-bottomed, 96-well plates (Thermo Labsystems, Franklin, MA, United States) to detect AI-2 activity. The CF collected from BB152 and DH5α cultures was used as a positive and negative control, respectively. Luminescence was measured using a Tecan GENios Plus microplate reader in luminescence mode (Tecan Austria GmbH, Grodig, Austria).

D-Ribose inhibition of BB152 and L-ZS9 AI-2 activity was also determined by using the BB170 reporter strain. D-Ribose (0, 10, 50, and 100 mM) was added to AB medium containing diluted BB170 culture (1:5000, v/v) with the CF of BB152 (1:10, v/v) or L-ZS9 (1:10, v/v). After incubation at 28°C for 5 h, AI-2 activity was detected as described above.

### Biofilm Formation Assay

Crystal violet (CV) staining was used to quantify biofilm formation of *L. paraplantarum* L-ZS9. Briefly, overnight culture of *L. paraplantarum* L-ZS9 was diluted to an optical density of 0.1 at 600 nm. Diluted culture (200 μL) was transferred to a 96-well plate (Corning, United States). Different concentrations of AI-2 (3.7, 18.5, 37, 185 μM), D-ribose (10, 50, and 100 mM) was added, and the mixture was incubated at 37°C for 36 h. The wells were washed gently three times with phosphate-buffered saline, stained with 0.1% CV for 30 min at room temperature, rinsed with distilled water, air-dried, and 100 μL 95% ethanol added to dissolve the CV. The absorbance at 595 nm was determined using a Synergy 2 microplate reader (Biotek, Winooski, VT, United States).

### Proteomic Analysis

*Lactobacillus paraplantarum* L-ZS9 treated with and without D-ribose (100 mM) was used for gel-based proteome analysis. Briefly, total-cell protein was extracted by trichloroacetic acid / acetone precipitation (Lakshman et al., 2008). Isolated protein was resuspended in lysis buffer (7 M urea, 2 M thiourea, 2%, w/v, CHAPS) and stored at -80°C until analysis. Protein concentration was determined by Bradford protein assay (Bio-Rad).

For electrophoresis, 400 μg of total protein was loaded onto immobilized pH-gradient strips (17 cm, non-linear pH 4-7; Bio-Rad). Isoelectric focusing and sodium dodecyl sulfate polyacrylamide gel electrophoresis were carried out as previously described (Wang et al., 2006). After electrophoresis, gels were stained with Colloidal Coomassie Brilliant Blue G-250, imaged using an ImageScanner (Amersham Biosciences), and analyzed by ImageMaster 2D Platinum 6.0 software (Wang et al., 2006). Protein spots with ≥1.5-fold change in abundance (*p* < 0.05) were subjected to protein identification.

The selected protein spots were excised, and in-gel digestion was performed with 0.01 μg/μL trypsin (Promega, Madison, WI, United States) in 25 mM ammonium bicarbonate. Tryptic peptides were dissolved in 0.5% (w/v) trifluoroacetic acid. Matrix-assisted laser desorption/ionization time-of-flight mass spectrometry and tandem time-of-flight/time-of-flight mass spectrometry were carried out on a 4800 Proteomics Analyzer (Applied Biosystems, United States) as described previously (Wang et al., 2006). Combined mass with mass/mass spectra was used to interrogate protein sequences in the NCBI database using the MASCOT database search algorithms (version 2.1, Matrix Science, London, United Kingdom). A protein was reported as identified if the Mascot score confidence interval > 95%. Functional analyses of the differentially expressed proteins were conducted using the Universal Protein Resource^2^ and the Kyoto Encyclopedia of Genes and Genomes^3^.

### Quantitative Real-Time PCR (qRT-PCR)

*Lactobacillus paraplantarum* L-ZS9 was cultured in MRS with D-ribose (100 mM) or AI-2 (18.5 μM) at 37°C to logarithmic phase. *L. paraplantarum* L-ZS9 cultured without AI-2 or D-ribose was used as a control. Total RNA was extracted with TRIzol (Invitrogen, United States) according to the manufacturer’s instructions. RNA quality was determined by measuring *A*_260_/*A*_280_ and *A*_260_/*A*_230_ and by gel electrophoresis. Isolated RNA was transcribed into single-stranded cDNA using a TUREscript 1st Strand cDNA Synthesis Kit (Aidlab, China). qRT-PCR was performed by using SYBR Green assay kit (Tiangen, China) and a 7500 Fast Real-Time PCR system (Applied Biosystems). Primers were designed by Primer 3 Input^4^. The *16S rRNA* gene was used as an internal reference. The relative expression of specific genes was calculated by using the 2^-ΔΔ*C*_T_^ method according to Livak and Schmittgen (2001).

### Construction of Plasmids and Bacterial Strains

Chromosomal DNA of *L. paraplantarum* L-ZS9 was isolated using a TIANamp Bacteria DNA Kit (Tiangen Biotech, Beijing). The genes *tuf, fba, gap, pgm, nfo, rib*, and *rpoN* were amplified from the chromosomal DNA by PCR and cloned into pMD18T vector (Takara, Dalian). The constructed *tuf, fba, gap, pgm, nfo, rib*, and *rpoN*-pMD18T vectors and the pMG76e vector (provided by Professor Shangwu Chen, China Agricultural University) were digested with Fastdigest enzymes *Xba*I and *Xho*I (Thermo, United States). The gene fragments were inserted into the linearized pMG76e plasmid using a Rapid DNA Ligation Kit (Thermo, United States), and the constructed vector was transformed into *E. coli* DH5α. The constructed plasmids harboring the genes of interest were identified by PCR and extracted by using a TIANpure Midi Plasmid Kit (Tiangen, China). Constructed pMG76e and empty pMG76e plasmids were electrotransformed into *L. paraplantarum* L-ZS9 competent cells, and recombinant strains were selected with erythromycin and confirmed by PCR.

### Biofilm Formation of Recombinant Strains

The growth of recombinant strains was analyzed by measuring the optical density at 600 nm to determine the effect of the expression the genes of interest on the growth of L-ZS9. Biofilm formation of recombinant strains over-expressing *tuf, fba, gap, pgm, nfo, rib*, and *rpoN* was measured by CV staining. Overnight cultures of recombinant strains and the wild-type strain were diluted in MRS without erythromycin to an optical density of 0.1 at 600 nm, and 200 μL of each suspension was transferred to a 96-well plate (Corning, NY, United States). After incubation at 37°C for 36 h, the biofilm formation of these strains was measured as described above.

### Statistical Analysis

The data were analyzed by one-way analysis of variance (ANOVA) using Graphpad Prism 5.0. Data are presented as means ± SEM. A *p*-value less than 0.05 was considered as significant.

## Results

### *L. paraplantarum* L-ZS9 Contains All the Genes Responsible for AI-2 Production But Not *sahH*

**FIGURE 1:**
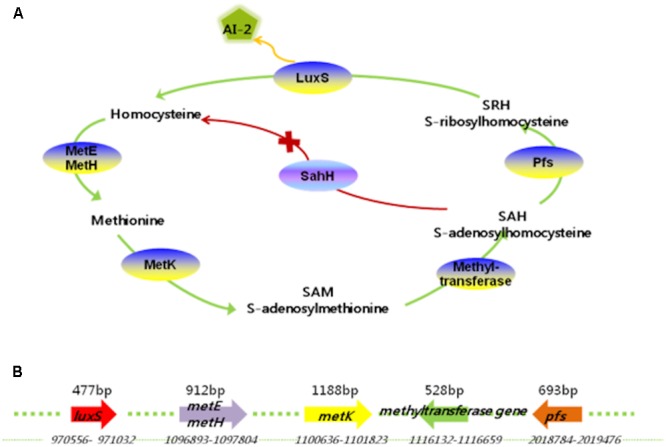
AI-2 production pathway **(A)** and sizes and locations of related genes **(B)** in *L. paraplantarum* L-ZS9.

Biosynthesis of AI-2 is part of the methionine catabolism cycle. MetE/MetH is responsible for transforming homocysteine into methionine. Methionine is then converted to *S*-adenosylmethionine (SAM) in a reaction catalyzed by MetK. Methyltransferase transforms SAM to SAH, which is converted to homocysteine through Pfs and LuxS, and AI-2 is produced (Schauder et al., 2001; Parveen and Cornell, 2011). Complete genome analysis showed that *L. paraplantarum* L-ZS9 contains all the genes involved in AI-2 production, including *pfs, luxS, metE*/*metH, metK*, and the methyltransferase gene, but *sahH* or its homologous gene, responsible for changing SAH to homocysteine directly, was not found (**Figure 1A**). These results indicated that *L. paraplantarum* L-ZS9 metabolized SAH to homocysteine through Pfs and LuxS, and produced AI-2 as a byproduct. The size and location of these genes are shown in **Figure 1B**.

### *L. paraplantarum* L-ZS9 Produced AI-2 When Cultured in Skim Milk Medium

**FIGURE 2:**
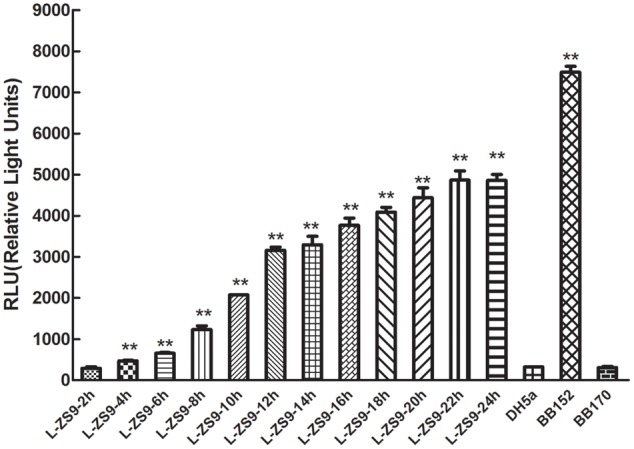
AI-2 activity in cell-free culture fluids (CFs) of *L. paraplantarum* L-ZS9. *V. harveyi* BB152 served as a positive control and *E. coli* DH5α as a negative control. Data are presented as mean ± SEM. *n* ≥ 3. ^∗^*p* < 0.05, ^∗∗^*p* < 0.01 compared with the negative control.

AI-2 production was measured by using the reporter strain *V. harveyi* BB170 in a bioassay with CFs of *L. paraplantarum* L-ZS9 cultured in skim milk medium. *V. harveyi* BB152 CF with confirmed AI-2 activity (7492.604 relative light units (RLUs)) was used as a positive control, *E. coli* DH5α CF with no AI-2 activity (321.805 RLUs) was used as a negative control, and *V. harveyi* BB170 with no CF (307.740 RLUs) was used as a blank control. AI-2 activity was detected in CFs prepared from *L. paraplantarum* L-ZS9 grown in skim milk medium and increased with increasing incubation time to reach a maximum value (4864.748 RLUs) after 22 h (**Figure 2**). These results suggested that *L. paraplantarum* L-ZS9 can produce AI-2 and that the AI-2 concentration depended on the incubation time.

### Inhibitory Effect of D-Ribose on AI-2 Activity of BB152 and L-ZS9

**FIGURE 3:**
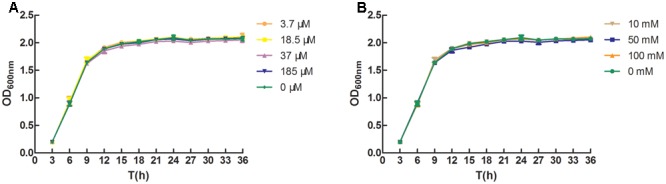
Effects of AI-2 **(A)** and D-ribose **(B)** on growth of *L. paraplantarum* L-ZS9. Growth of the strain in the absence of AI-2 or D-ribose served as a control. Data are presented as mean ± SEM. *n* ≥ 3. ^∗^*p* < 0.05, ^∗∗^*p* < 0.01 compared with the control.

**FIGURE 4:**
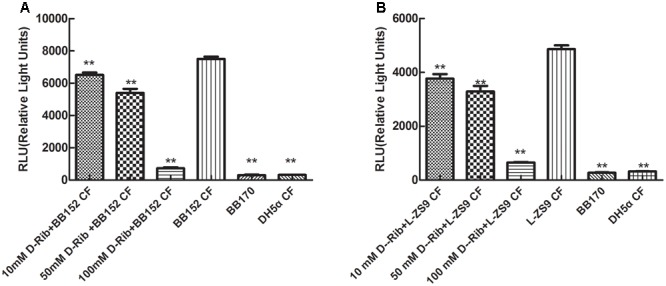
Effect of D-ribose on AI-2 activity of *V. harveyi* BB152 **(A)** and *L. paraplantarum* L-ZS9 **(B)**. *E. coli* DH5α served as a negative control. Data are presented as mean ± SEM. *n* ≥ 3. ^∗^*p* < 0.05, ^∗∗^*p* < 0.01 compared with the positive control.

As shown in **Figures 3A,B**, exogenous AI-2 and D-ribose had no influence on growth of *L. paraplantarum* L-ZS9. D-Ribose significantly inhibited the AI-2 activity of *V. harveyi* BB152 and *L. paraplantarum* L-ZS9 in a dose-dependent manner (**Figures 4A,B**); 100 mM D-ribose inhibited AI-2 activity of *V. harveyi* BB152 and *L. paraplantarum* L-ZS9 to about 0.10-fold and 0.13-fold, respectively. The result indicated that D-ribose could be used as a QSI against AI-2 activity of L-ZS9.

### Inhibitory Effect of D-Ribose on Biofilm Formation of L-ZS9 Induced by AI-2

**FIGURE 5:**
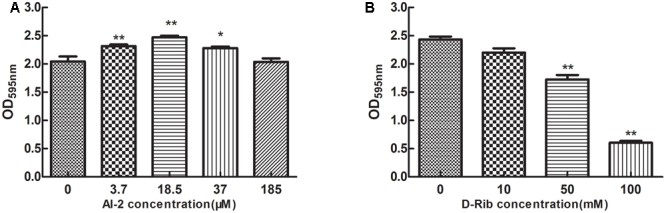
Effect of AI-2 **(A)** and D-ribose **(B)** on biofilm formation of *L. paraplantarum* L-ZS9. The biofilm formation of the strain in the absence of AI-2 or D-ribose served as a control. Data are presented as mean ± SEM. *n* ≥ 3. ^∗^*p* < 0.05, ^∗∗^*p* < 0.01 compared with the control.

**Figure 5A** shows that 3.7–37.0 μM AI-2 increased biofilm formation of *L. paraplantarum* L-ZS9, which peaked at 18.5 μM AI-2. Different concentrations of AI-2 showed different effects on biofilm formation of L-ZS9. The effect of AI-2 on biofilm formation was not dose-dependent; the highest biofilm formation was observed when cultures were treated with 18.5 μM AI-2, and decreased with increasing concentration of AI-2. On the contrary, D-ribose inhibited the biofilm formation of L-ZS9 in a dose-dependent manner, and 100 mM D-ribose inhibited the biofilm formation of L-ZS9 to about 0.25-fold compared with the control (**Figure 5B**).

### Protein Identification in *L. paraplantarum* L-ZS9 Treated with or without D-Ribose

**FIGURE 6:**
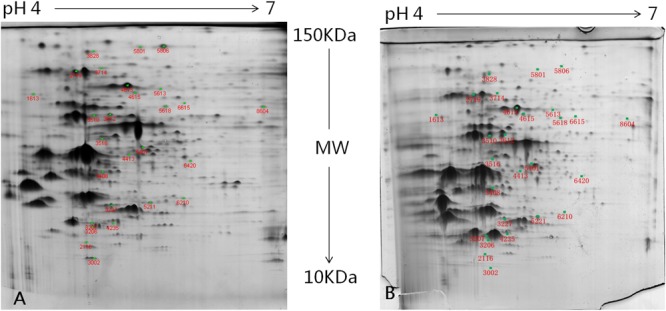
Two-dimensional electrophoresis of the total-cell proteins of *L. paraplantarum* L-ZS9 treated without **(A)** or with D-ribose **(B)**. Differentially expressed protein spots of cells treated with or without D-ribose were numbered according to the numbering in **Table 1**.

To profile the impact of D-ribose on protein expression, *L. paraplantarum* L-ZS9 was cultured with or without D-ribose. The proteomes were comparatively studied by gel-based proteomic analysis. Preliminary 2D gel electrophoresis analysis with a wide pH gradient (pH 3–10) showed that the majority of proteins had their isoelectric point (pI) in the acidic region (data not shown). Therefore an immobilized pH-gradient strip with a pH gradient of 4–7 was used in this study. As shown in **Figure 6**, 27 differentially expressed proteins were detected (*P* < 0.05) (details of biological replicates are provided in the Supplementary Material). Among them, 15 proteins showed up-regulation and 12 proteins showed down-regulation by at least 1.5-fold following D-ribose treatment. The molecular weight (MW) and pI of these proteins are listed in **Table 1**. Functionally, these differentially expressed proteins were classified into six groups that related to carbon and carbohydrate metabolism, amino acid metabolism, nucleotide transport and metabolism, fatty acid metabolism, transcription and translation, and other functions. Function classification of these proteins suggested that D-ribose may inhibit biofilm through multiple mechanisms, which include regulating translation and transcriptional activity, carbohydrate and energy metabolism, amino acid synthesis, and protein and enzyme secretion.

**Table 1:** Functional classification of differentially expressed proteins in cells of *L. paraplantarum* L-ZS9 treated with or without D-ribose.

Spot no.	Accession no.	Gene	Protein name	Protein MW	Protein pI	Fold change^a^
**Carbon metabolism and carbohydrate metabolism**		
2719	gi| 380033724	*pgm*	Phosphoglycerate mutase family protein	26071.3	4.94	0.56
3510	gi| 545603975	*rib*	Ribokinase	32080.3	5.02	3.24
3612	gi| 545603975	*rib*	Ribokinase	32080.3	5.02	3.01
4613	gi| 380031375	*fba*	Fructose-bisphosphate aldolase	30901.6	5.07	0.47
5618	gi| 254555821	*gap*	Glyceraldehyde-3-phosphate dehydrogenase	36415.6	5.3	0.54
5613	gi| 380031375	*fba*	Fructose-bisphosphate aldolase	30901.6	5.07	2.34
3206	gi| 545606863	*ptk*	Phosphoketolase	88690.1	5.05	5.12
**Amino acid metabolism**		
6210	gi| 545603889	*asnB*	Asparagine synthase	73077.3	5.72	0.21
6420	gi| 545606144	*murD*	UDP-*N*-acetylmuramoyl-L-alanyl-D-glutamate synthetase	49993.6	5.75	1.67
**Nucleotide transport and metabolism**		
3828	gi| 254556519	*gmk*	Guanylate kinase	23445.7	4.99	2.33
1613	gi| 545605966	*rihC*	Ribonucleoside hydrolase RihC	33030.6	4.67	2.18
3516	gi| 545605039	*guaC*	Guanosine 5′-monophosphate oxidoreductase	39843.3	5.21	0.3
4413	gi| 544928185	*purA*	Adenylosuccinate synthetase	47207.3	5.25	0.46
6615	gi| 545604536	*nfo*	Endonuclease IV	31603.1	5.65	2.1
***Amino acid metabolism***		
4615	gi| 545605392	*smp*	*S*-malonyltransferase	33242.1	5.59	1.77
5211	gi| 513840005	*lai*	Linoleic acid isomerase	64204.6	5.37	1.74
**Transcription and translation**		
2116	gi| 545606085	*tuf*	Elongation factor Tu	43364.1	4.95	0.1
3002	gi| 550695603	*rpoB*	DNA-directed RNA polymerase subunit beta	131576.3	4.87	0.02
3207	gi| 545606497	*fusA*	Elongation factor G	76921.7	4.82	9.91
3227	gi| 254556435	*thrS*	Threonyl-tRNA synthetase	73773	5.1	0.43
3408	gi| 545606490	*serS*	Seryl-tRNA synthetase	48103.2	5.15	2.2
5801	gi| 545605166	*rpoN*	Sigma 54 factor, RpoN	21724.2	5.47	0.14
5806	gi| 545605166	*rpoN*	Sigma 54 factor, RpoN	21724.2	5.47	0.03
**Other**		
3714	gi| 254556461	*rrg*	Response regulator	26356	5.06	0.55
4235	gi| 545605165	*secA*	Preprotein translocase subunit SecA	89545.2	5.16	3.13
5401	gi| 545603952	*nirD*	Assimilatory nitrite reductase, subunit	44407.8	5.51	2.2
8604	gi| 545604158	*adh*	Aryl-alcohol dehydrogenase family enzyme	36955.8	6.71	2.03

*^a^D-Ribose-treated vs. untreated cells.*

### Effects of D-Ribose and AI-2 on Gene Transcription of *L. paraplantarum* L-ZS9

**FIGURE 7:**
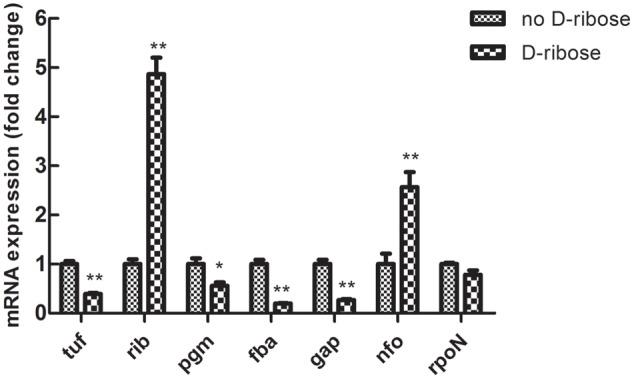
Effects of D-ribose (100 mM) on mRNA expression of *tuf, fba, gap, pgm, nfo, rib*, and *rpoN* genes in *L. paraplantarum* L-ZS9. The mRNA expression of these genes in the absence of D-ribose served as a control. Data are presented as mean ± SEM. *n* ≥ 3. ^∗^*p* < 0.05, ^∗∗^*p* < 0.01 compared with the control.

As shown in **Figure 7**, D-ribose changed the transcription of the *tuf, fba, gap, pgm, nfo, rib*, and *rpoN* genes of *L. paraplantarum* L-ZS9, which was consistent with the effect of D-ribose on their protein levels. These genes were selected from those encoding the differentially expressed proteins of the above 2D polyacrylamide gel electrophoresis results based on their relationship to QS, which has been reported in previous research (Wolfe et al., 2004; Da et al., 2008; Kiedrowski et al., 2011; Iyer and Hancock, 2012; Sahu et al., 2012; Zhou et al., 2012; Hao et al., 2013; Li Z. et al., 2015; Rajendran et al., 2015). D-Ribose inhibited the transcription of *tuf, pgm, fab, gap*, and *rpoN* to about 0.39-fold, 0.56-fold, 0.20-fold, 0.27-fold, and 0.78-fold, respectively, while it increased the transcription of *nfo* and *rib* to about 2.57-fold and 4.87-fold, respectively.

**FIGURE 8:**
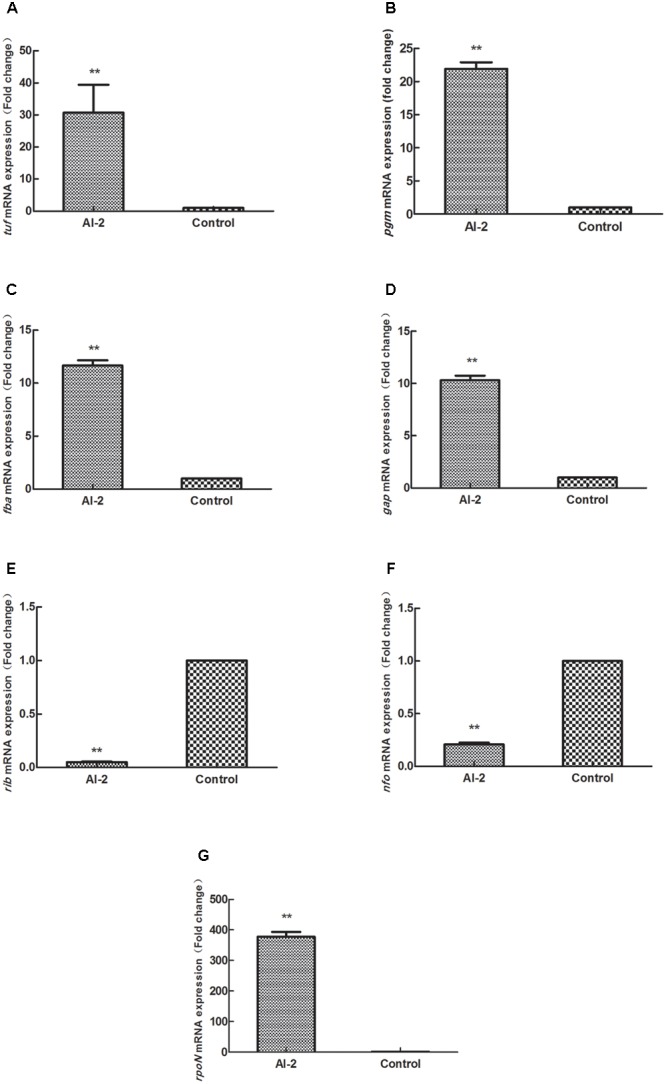
Effects of AI-2 (18.5 μM) on mRNA expression of **(A–G)** *tuf, fba, gap, pgm, nfo, rib, and rpoN* genes in *L. paraplantarum* L-ZS9. The mRNA expression of these genes in the absence of AI-2 served as a control. Data are presented as mean ± SEM. n ≥ 3. ^∗^*p* < 0.05, ^∗∗^*p* < 0.01 compared with the control.

D-Ribose produced inhibition of AI-2 activity and biofilm formation of L-ZS9, which was induced by AI-2. Thus, the effect of AI-2 on gene expression was investigated. As shown in **Figure 8**, exogenous AI-2 changed the transcription of *tuf, fba, gap, pgm, nfo, rib*, and *rpoN* in *L. paraplantarum* L-ZS9. Opposite to the effect of D-ribose, AI-2 increased transcription of *tuf, pgm, fab, gap* and *rpoN* to about 30-fold, 20-fold, 11-fold, 10-fold, and 378-fold, respectively. And AI-2 decreased the transcription of gene *nfo* and *rib* to about 0.21-fold and 0.5-fold, respectively. These results showed that AI-2 and D-ribose had opposite effects on the expression of *tuf, fba, gap, pgm, nfo, rib*, and *rpoN*.

### *tuf, fba, gap, pgm, nfo, rib*, and *rpoN* Regulated Biofilm Formation of *L. paraplantarum* L-ZS9

**FIGURE 9:**
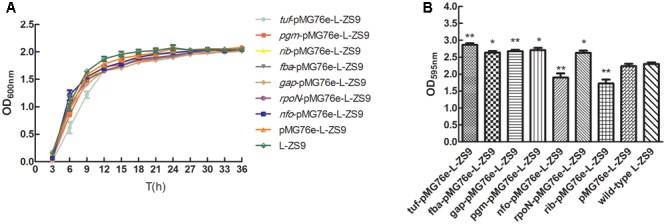
Growth curves **(A)** and biofilm formation **(B)** of recombinant *L. paraplantarum* L-ZS9 strains. The wild-type strain *L. paraplantarum* L-ZS9 served as a control. Data are presented as mean ± SEM. *n* ≥ 3. ^∗^*p* < 0.05, ^∗∗^*p* < 0.01 compared with the control.

To validate the effects of *tuf, fba, gap, pgm, nfo, rib*, and *rpoN* gene expression on biofilm formation of *L. paraplantarum* L-ZS9, recombinant strains over-expressing these genes were constructed and their biofilm formation abilities were compared with those of the wild-type strain. Over-expression of these genes had no significant effect on the growth of *L. paraplantarum* L-ZS9 (**Figure 9A**). As shown in **Figure 9B**, over-expression of *tuf, pgm, fba, gap*, and *rpoN* increased biofilm formation and over-expression of *nfo* and *rib* inhibited biofilm formation of *L. paraplantarum* L-ZS9 significantly.

## Discussion

The LuxS/AI-2 QS system has been found in a variety of bacteria, including both Gram-negative and Gram-positive species. The non-species-specific signal AI-2, which acts as a universal QS signal for interaction between bacterial species, is a byproduct of SAM metabolism with LuxS as the key enzyme. Biosynthesis of AI-2 involves a three-step reaction, which is part of the methionine catabolism cycle. The first step is the removal of a methyl group from SAM, which is catalyzed by SAM-dependent methyltransferases. The resulting product, SAH, is converted to *S*-ribosylhomocysteine by the enzyme Pfs (Parveen and Cornell, 2011). *S*-ribosylhomocysteine is hydrolyzed to 4,5-dihydroxy-2,3-pentanedione by LuxS (Schauder et al., 2001). 4,5-Dihydroxy-2,3-pentanedione is further auto-hydrolyzed to form AI-2. SAH exists widely in organisms and is transformed to homocysteine through one-step or two-step conversion. In most bacteria, SAH is metabolized using two-step conversion by the enzymes Pfs and LuxS with concomitant AI-2 production. However, in some prokaryotes and eukaryotes, SAH can be metabolized by one-step conversion using SAH hydrolase (SahH) with no AI-2 production. In this study, genome sequence analysis suggested that L-ZS9 degrades SAH via the enzymes Pfs (MTAN) and LuxS to produce AI-2, without SAH hydrolase (SahH) (**Figure 1**). In addition, AI-2 activity in CFs of L-ZS9 was determined. AI-2 activity is usually detected by using the reporter strain *V. harveyi* BB170 (sensor^1-^ sensor^2+^), which only recognizes AI-2 (Vilchez et al., 2007). However, a high concentration of glucose and acidic pH can inhibit AI-2 activity (DeKeersmaecker and Vanderleyden, 2003; Turovskiy and Chikindas, 2006; Cuadra et al., 2016). Therefore, as for lactic acid bacteria, AI-2 activity detection is difficult under traditional cultural conditions and CF preparation. As a consequence, there was no AI-2 activity detected in *L. paraplantarum* L-ZS9 when the strain was cultured in MRS broth (data not shown). However, AI-2 activity was detected when *L. paraplantarum* L-ZS9 was cultured in skim milk medium and CFs were adjusted to pH 7.0 (**Figure 2**). This preparation method can also be used for AI-2 activity detection of other lactic acid bacteria. According to these results, *L. paraplantarum* L-ZS9 may have the capacity to communicate with itself and other strains by AI-2. Therefore, LuxS/AI-2 QSI could play an important role in interfering with the social behavior of L-ZS9.

Ribose has been reported to be used as a QSI of the LuxS/AI-2 QS system by competing for AI-2 receptor (James et al., 2006; Armbruster et al., 2011). Our data showed that D-ribose could inhibit AI-2 activity of L-ZS9, suggesting that it can be used as a QSI of the LuxS/AI-2 QS system in L-ZS9. LuxS/AI-2 QS regulates biofilm formation of many bacteria such as *Streptococcus mitis, Pseudomonas aeruginosa* PAO1, *Eikenella corrodens, Candida albicans, Riemerella anatipestifer, Aggregatibacter actinomycetemcomitans*, and *Bifidobacteria* (Shao et al., 2007; Karim et al., 2013; Bachtiar et al., 2014; Christiaen et al., 2014; Han et al., 2015; Li H. et al., 2015; Wang et al., 2016). However, limited studies have been conducted on the regulation of biofilm formation in *Lactobacillus* by the AI-2/LuxS QS system. It has been reported that *luxS* of *Lactobacillus rhamnosus* GG plays a central metabolic role in biofilm formation (Lebeer et al., 2007a). *Lactobacillus* uses the AI-2 signal to respond to environment stress and to regulate growth and metabolism (Lebeer et al., 2007b, 2008; Moslehi-Jenabian et al., 2009; Yeo et al., 2015). The present study showed that AI-2 increased biofilm formation of *L. paraplantarum* L-ZS9 (**Figure 5A**). D-Ribose has been reported to inhibit co-culture biofilm formation of *P. aeruginosa* PAO1 and *Streptococcus mitis* significantly (Wang et al., 2016). Ribose could also inhibit streptococcal biofilm formation (Lee et al., 2015). In this study, D-ribose was confirmed to significantly inhibit the biofilm formation of *L. paraplantarum* L-ZS9 as well (**Figure 5B**). These results suggest that a universal QSI such as ribose could inhibit biofilm formation of non-pathogenic and beneficial microorganisms, which must be taken into consideration when a universal QSI is used for against pathogens.

**FIGURE 10:**
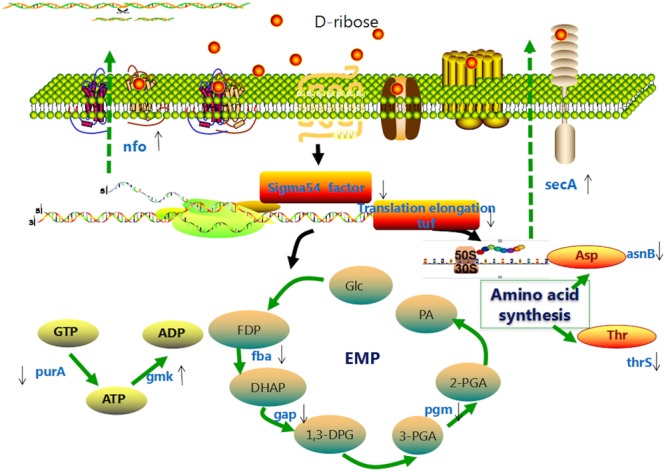
Hypothetical model of the effect of D-ribose on *L. paraplantarum* L-ZS9 cells. Glc, glucose; FDP, fructose-1,6-diphosphate; DHAP, dihydroxyacetone phosphate; 1,3-DPG, 1,3-diphosphoglyceric acid; 3-PGA, 3-phosphoglyceric acid; 2-PGA, 2-phosphoglyceric acid; PA, pyruvic acid; EMP, glycolysis.

To investigate the inhibition mechanism of D-ribose in regulating biofilm formation, relevant genes were firstly identified by comparative proteomic analysis. According to the proteomic analysis and previous research (Wolfe et al., 2004; Da et al., 2008; Kiedrowski et al., 2011; Iyer and Hancock, 2012; Sahu et al., 2012; Zhou et al., 2012; Hao et al., 2013; Li H. et al., 2015; Rajendran et al., 2015), D-ribose may function through gene regulation as shown in **Figure 10**. The influence of D-ribose and AI-2 on gene transcription (*tuf, fba, gap, pgm, nfo, rib*, and *rpoN*) was then further evaluated by qRT-PCR. Our data showed that D-ribose, a LuxS/AI-2 QS system QSI, regulated the expression of *tuf, fba, gap, pgm, nfo, rib*, and *rpoN* in an opposite fashion to AI-2. Biofilm assays of recombinant strains further indicated that the overexpression of *fba, gap*, and *pgm* could increase the biofilm formation of L-ZS9. The *fba, gap*, and *pgm* genes play key roles in the glycolytic pathway (EMP), which is important for energy metabolism. *fba* is responsible for transformation of frucose-1,6-diphosphate to dihydroxy acetone phosphate, which is transformed to 1,3-diphosphoglycerate by *gap*, and *pgm* transforms 3-phosphoglyceride to 2-phosphoglycerate. EMP has been reported to play key roles in biofilm formation and procession of *Saccharomyces cerevisiae* (Li H. et al., 2015). Proteins involved in EMP were indicated to be up-regulated in biofilm cells of *Listeria monocytogenes* compared with planktonic cells (Zhou et al., 2012). Our data also showed that EMP in L-ZS9 was positively correlated with biofilm formation. In addition, EMP may play roles in sugar metabolism as well. Previous studies elucidated that extracellular polysaccharide (EPS) is important in biofilm formation (Blackledge et al., 2013). Stoodley et al. (2002) reported the possible role of QS as a signal transduction system to initiate the production of alginate and possibly other types of EPS in *P. aeruginosa*. Based on these studies, it is logical to deduce that AI-2 and D-ribose could affect biofilm formation of *L. paraplantarum* L-ZS9 by regulating EPS synthesis.

D-Ribose and AI-2 increased and decreased the expression of the *nfo* gene, respectively, which encodes an endonuclease. Extracellular DNA is one of the important components of biofilm matrix, which could be degraded by various nucleases. DNase is able to break biofilm formation of Gram-positive and Gram-negative bacteria rapidly (Nijland et al., 2010). It was reported that *comEB* regulated biofilm formation of *Staphylococcus lugdunensis* by influencing production of DNA (Rajendran et al., 2015). As for *Acinetobacter baumannii* AIIMS 7, extracellular DNA was degraded and biofilm was decreased to 59.41% after treatment with DNase I (Sahu et al., 2012). In *Staphylococcus aureus* and *Neisseria gonorrhoeae*, nuclease modulated biofilm formation by regulating extracellular DNA (Kiedrowski et al., 2011; Steichen et al., 2011). Expression of extracellular endonucleases also influenced the biofilm formation of *Shewanella oneidensis* MR-1 (Godeke et al., 2011), and DNase 1L2 suppressed biofilm formation of *P. aeruginosa* and *Staphylococcus aureus* (Eckhart et al., 2007). Similar to other research, over-expression of endonuclease also inhibited biofilm formation of L-ZS9 in our study.

Our data also showed that the biofilm formation of strain *tuf*-76e-L-ZS9 was increased compared with the wild-type strain. The *tuf* gene encodes elongation factor thermo unstable (EF-Tu) and functions in translation elongation, protein folding, and protection from stress (Caldas et al., 1998). Although no research has reported that EF-Tu modulates biofilm formation directly, it has been identified as an adherence-related factor that may affect biofilm formation. EF-Tu plays important roles in adherence of *Lactobacillus*, such as *Lactobacillus acidophilus* ATCC 4356, *Lactobacillus plantarum* 423, and *Lactobacillus johnsonii* NCC533 (La1) (Ramiah et al., 2008, 2009; Dhanani and Bagchi, 2013). This is the first time to our knowledge that EF-Tu has been reported to modulate biofilm formation directly, which may be due to regulation of the initial adherence of L-ZS9. Over-expression of *rpoN* that encodes σ54 factor also promoted the biofilm formation of L-ZS9. The σ54 factor is structurally and functionally distinct from all other σ factors and is required for initiation of transcription (Shingler, 2011). In addition, σ54 regulates utilization of alternative carbon sources, detoxification systems, assembly of motility organs, and production of extracellular alginate (Maxson and Darwin, 2006). Although the role of σ54 remains unclear in *Lactobacillus*, it has been reported that σ54 correlates with biofilm formation directly or indirectly in other bacteria (Belik et al., 2008; Saldias et al., 2008; Iyer and Hancock, 2012; Hao et al., 2013). For example, σ54 controls motility, biofilm formation, and colonization in *Vibrio fischeri* (Wolfe et al., 2004; Yip et al., 2005) and regulates genes involved in type I and type IV pili biogenesis to influence the biofilm formation of *Xylella fastidiosa* (Da et al., 2008). Based on the results of these studies, σ54 may play similar roles to regulate biofilm formation of L-ZS9. In addition, it was identified that *rib* also regulated biofilm formation of L-ZS9, and the underlying mechanism still needs to be further investigated. By exploring the effects of D-ribose on L-ZS9, this study identified the importance of serious consideration of LuxS/AI-2 QSI application due to its effects on non-pathogenic or beneficial bacteria. Modification of specific genes (for example genes identified in this research) should be conducted in order to protect these organisms from the QSI.

## Conclusion

*Lactobacillus paraplantarum* L-ZS9 contains key genes related to the LuxS/AI-2 QS system and is able to produce AI-2. D-Ribose can be used as a QSI of L-ZS9 and inhibits biofilm formation of L-ZS9 in contrast to exogenous AI-2 by regulating expression of the *tuf, fba, gap, pgm, nfo, rib*, and *rpoN* genes. This research provides new information about regulation mechanisms of the LuxS/AI-2 QS system in *Lactobacillus* and will be useful for determining the reasonable application of QSI.

## Author Contributions

LL and PL designed the experiments. LL, RW, and JZ performed the experiments. LL, NS, and PL analyzed the results and wrote the manuscript.

## Conflict of Interest Statement

The authors declare that the research was conducted in the absence of any commercial or financial relationships that could be construed as a potential conflict of interest.

## Footnotes

1 http://primer3.ut.ee/

2 http://www.ebi.ac.uk/uniprot/

3 http://www.genome.jp/kegg/

4 http://primer3.ut.ee/
