# Supplementary figures and images for: D-Ribose Interferes with Quorum Sensing to Inhibit Biofilm Formation of Lactobacillus paraplantarum L-ZS9
Source: Front Microbiol. 2017 Sep 26;8:1860. doi: 10.3389/fmicb.2017.01860 (PMC5622935; doi:10.3389/fmicb.2017.01860)

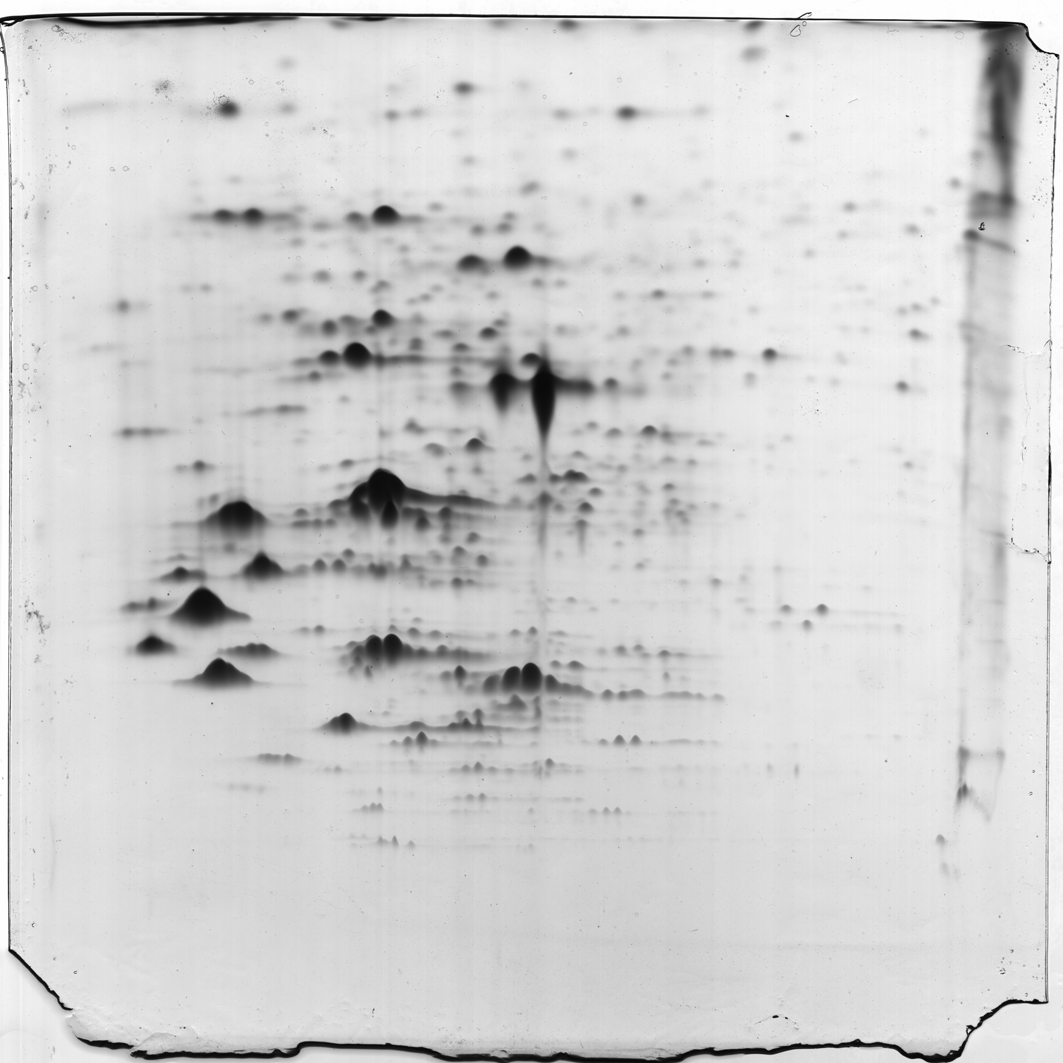

Supplement: Supplementary file 1 [file Image_1.TIF]

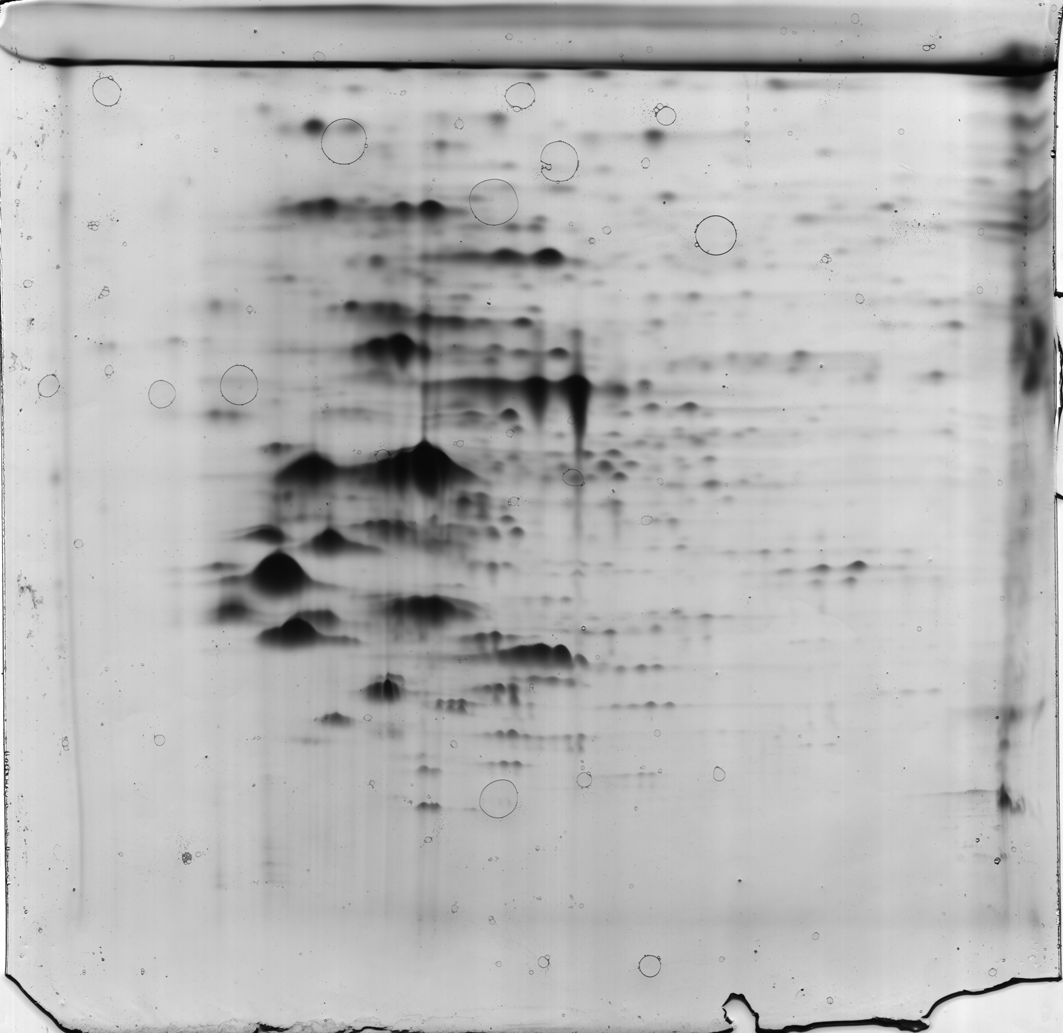

Supplement: Supplementary file 2 [file Image_2.TIF]

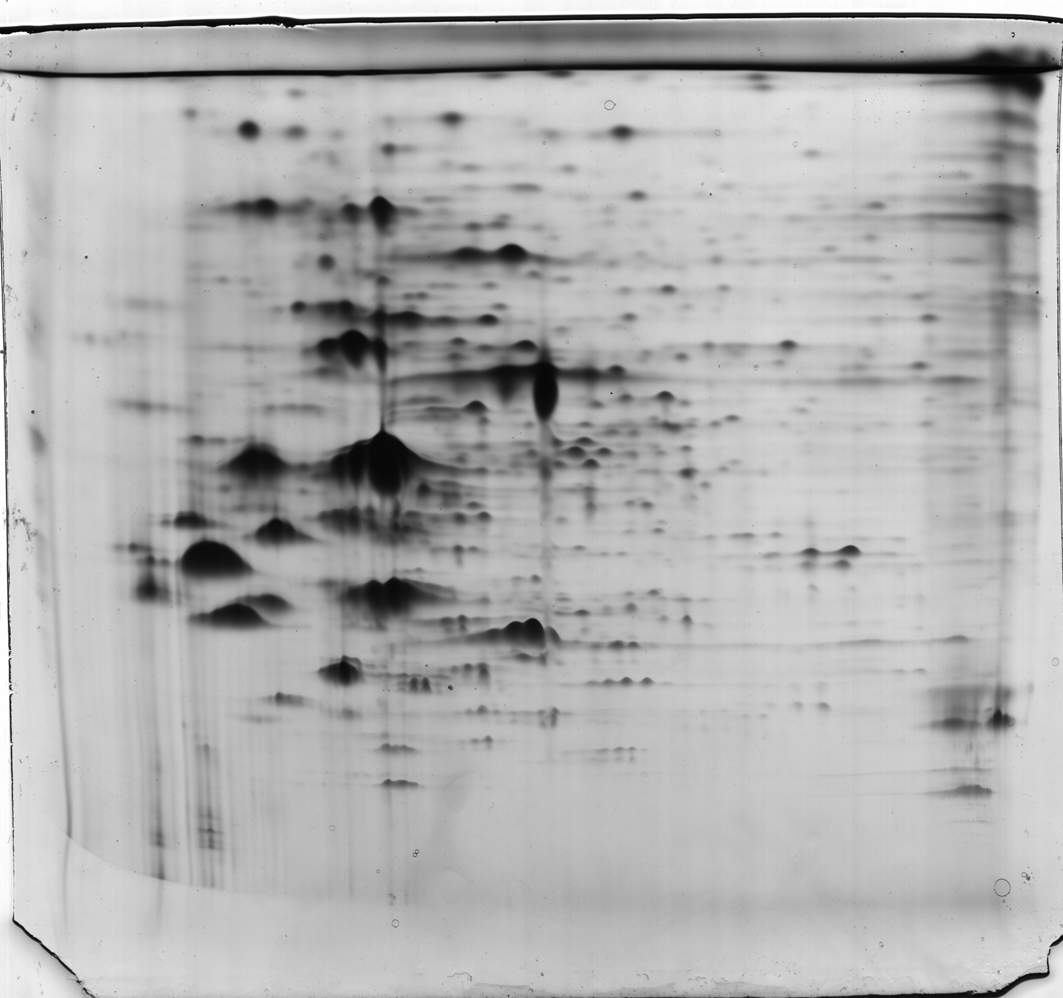

Supplement: Supplementary file 3 [file Image_3.TIF]

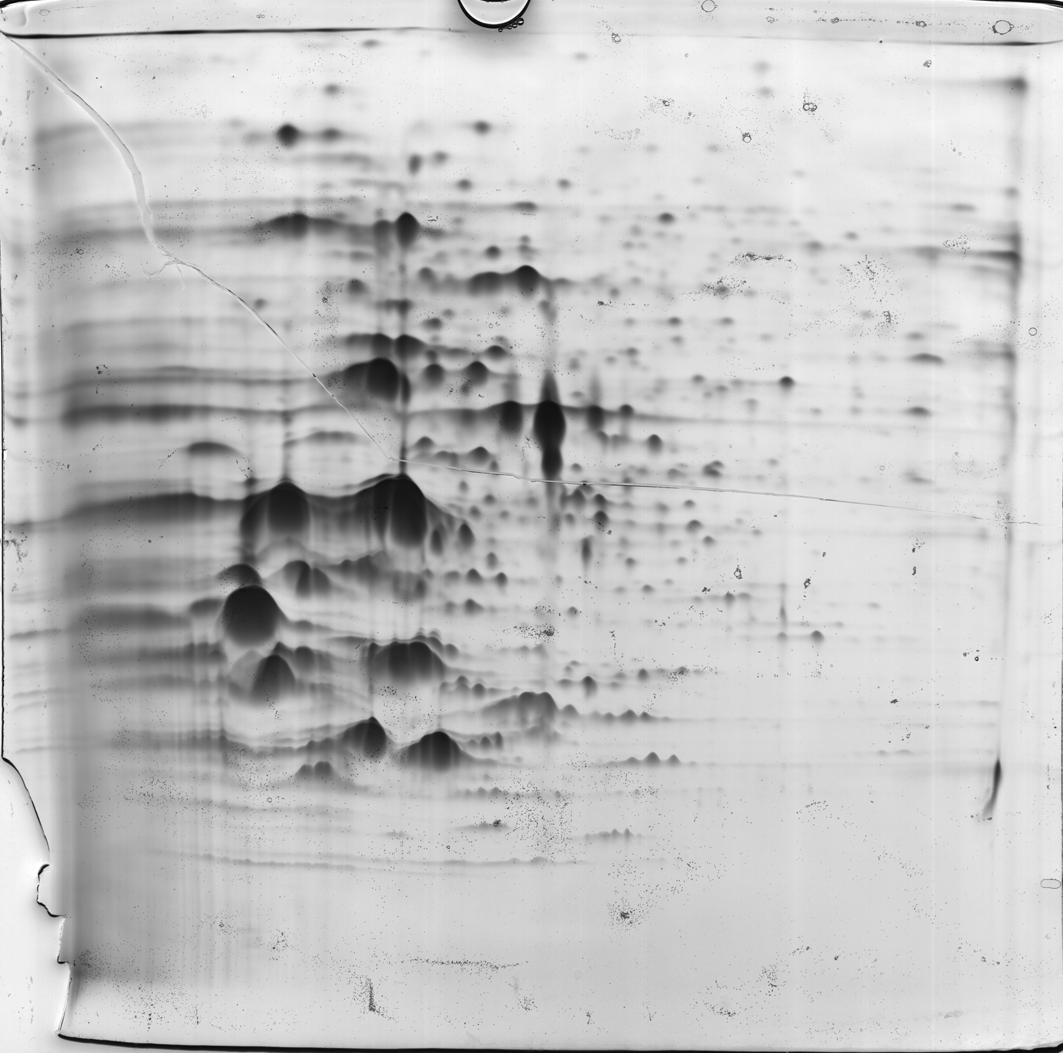

Supplement: Supplementary file 4 [file Image_4.TIF]

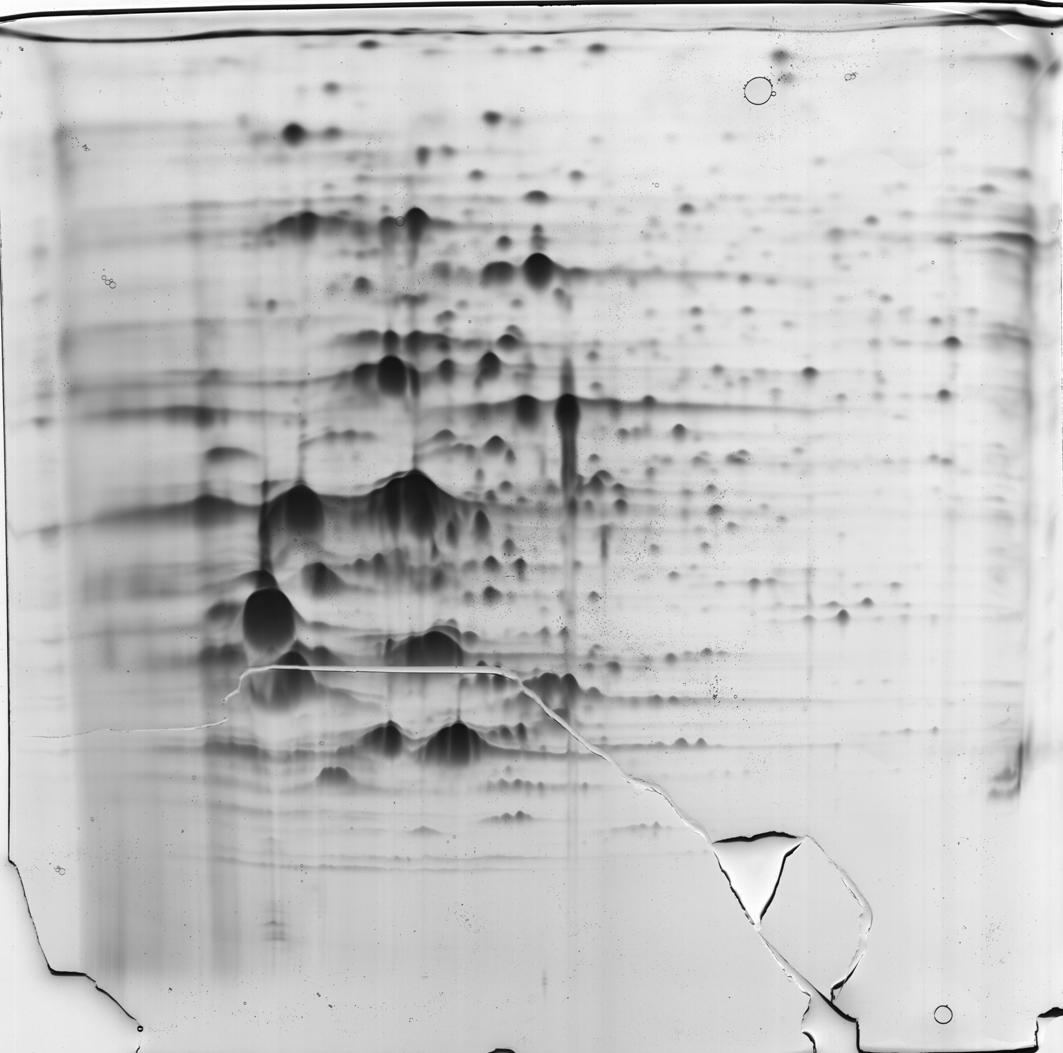

Supplement: Supplementary file 5 [file Image_5.TIF]

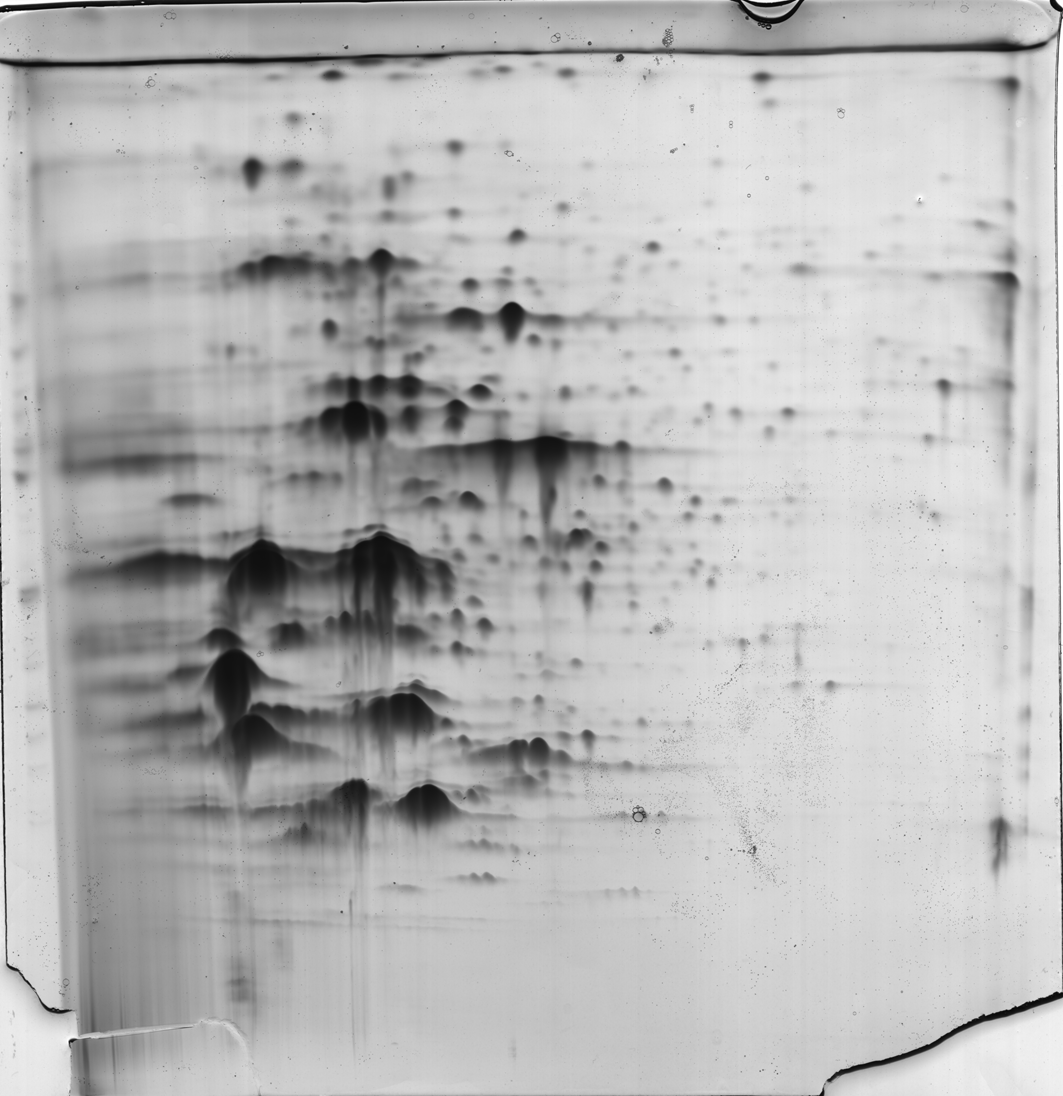

Supplement: Supplementary file 6 [file Image_6.TIF]

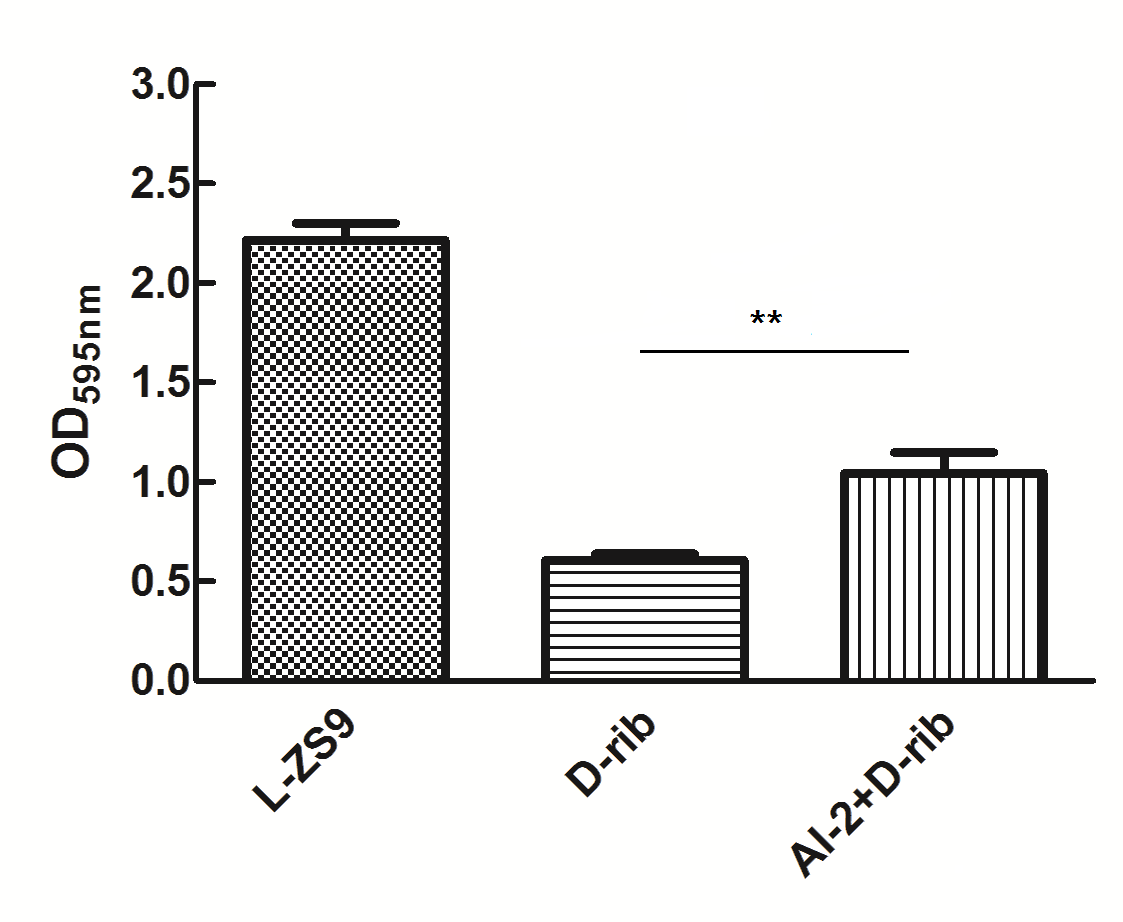

Supplement: Supplementary file 7 [file Image_7.TIF]
